# Pressure Induced Enhancement of Superconductivity in LaRu_2_P_2_

**DOI:** 10.1038/srep24479

**Published:** 2016-04-18

**Authors:** Baoxuan Li, Pengchao Lu, Jianzhong Liu, Jian Sun, Sheng Li, Xiyu Zhu, Hai-Hu Wen

**Affiliations:** 1 National Laboratory of Solid State Microstructures and Department of Physics, Collaborative Innovation Center of Advanced Microstructures, Nanjing University, Nanjing 210093, China

## Abstract

To explore new superconductors beyond the copper-based and iron-based systems is very important. The Ru element locates just below the Fe in the periodic table and behaves like the Fe in many ways. One of the common thread to induce high temperature superconductivity is to introduce moderate correlation into the system. In this paper, we report the significant enhancement of superconducting transition temperature from 3.8 K to 5.8 K by using a pressure only of 1.74 ± 0.05 GPa in LaRu_2_P_2_ which has an iso-structure of the iron-based 122 superconductors. The *ab-initio* calculation shows that the superconductivity in LaRu_2_P_2_ at ambient pressure can be explained by the McMillan’s theory with strong electron-phonon coupling. However, it is difficult to interpret the enhancement of *T*_*c*_ versus pressure within this picture. Detailed analysis of the pressure induced evolution of resistivity and upper critical field *H*_*c2*_*(T)* reveals that the increase of *T*_*c*_ with pressure may be accompanied by the involvement of extra electron-boson interaction. This suggests that the Ru-based system has some commonality as the Fe-based superconductors.

The transition metal compounds exhibit extremely interesting and rich physics due to the close energies concerning the charge, spin and orbital dynamics^1^,^2^. Usually the band width *t* is narrow in the *3d* transition metals, like Cu, Fe etc., therefore, compared with the electron repulsion energy (the so-called Hubbard *U*, about 1–5 eV), *t/U* is small and the correlation is quite strong. One of the consequence of this strong correlation is the formation of the magnetic state and possibly the local Cooper pairing^3^,^4^,^5^,^6^,^7^,^8^. In some *4d* transition metal compounds, the larger bandwidth makes the charge carriers more mobile against the strong Coulomb effect, yielding many exotic properties. The Ru element is a typical *4d* transition metal element which locates just below the Fe element in the periodic table and exhibits many appealing features. For example, *p*-wave superconductivity may exist in the Sr_2_RuO_4_ compound^9^,^10^,^11^. The LaRu_2_P_2_ has long been known to be a superconductor^12^,^13^,^14^ with *T*_*c*_ ≈ 3.8 K. Interestingly this compound has a similar structure as the parent phase BaFe_2_As_2_ of many iron-based superconductors in the 122 family^15^,^16^,^17^,^18^. Therefore it is very interesting to investigate what is the superconducting mechanism in this Ru-based 122 system, and check whether there is any clue of correlation effect in this system, which is crucial for copper-based and iron-based superconductors^3^,^19^,^20^. Here we report the pressure induced enhancement of superconductivity in a LaRu_2_P_2_ single crystal.

## Results

**Figure 1:**
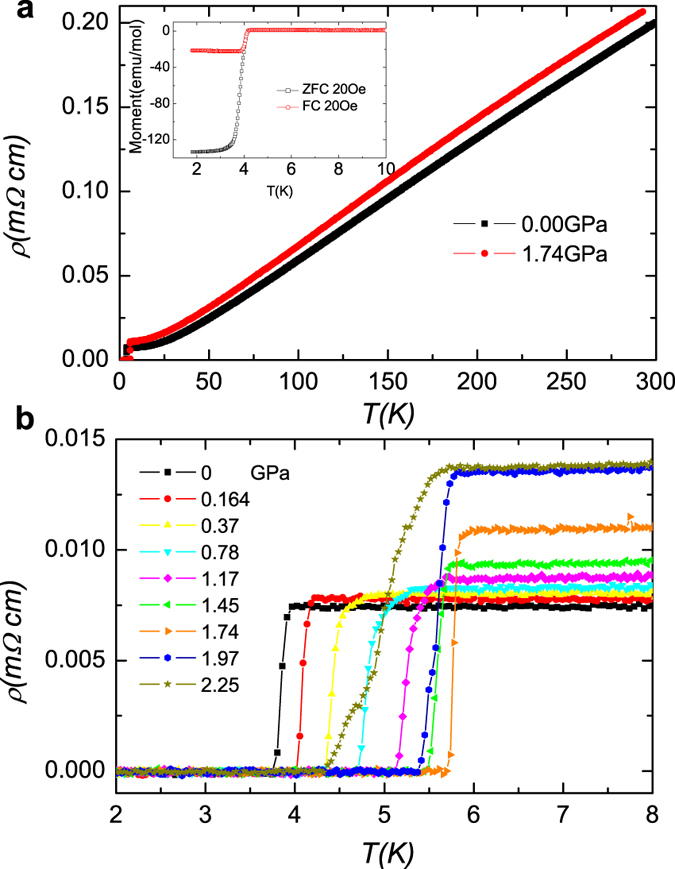
Pressure induced evolution of superconducting transitions. (**a**) Temperature dependence of electrical resistivity for the LaRu_2_P_2_ single crystal in the temperature range 2 K to 300 K as measured under ambient pressure and 1.74 ± 0.05 GPa, shown by the black square and red circle symbols, respectively. The inset shows the temperature dependence of dc magnetic susceptibility of the sample as measured at an applied magnetic field of 20 Oe at ambient pressure. Both the magnetic susceptibility measured in zero-field-cooled (ZFC) and field-cooled (FC) modes are shown. (**b**) Temperature dependence of electrical resistivity for the LaRu_2_P_2_ single crystal under various pressures from ambient to 2.25 ± 0.05 GPa.

In Fig. 1a we show the temperature dependence of resistivity of the LaRu_2_P_2_ single crystal at ambient pressure and 1.74 GPa. Details about the growth and characterization of the crystal are given in Methods and Supplementary Information. It is clear that the residual resistivity ratio *RRR* = *ρ*(300 K)/*ρ*(5 K) = 27.0 is quite large, this indicates the cleanness of the crystal. The inset of Fig. 1a shows the magnetic susceptibility near the superconducting transition at a field of 20 Oe with the zero-field-cooled (ZFC) and field-cooled (FC) mode. Fig. 1b presents the temperature dependence of resistivity under different pressures from ambient to 2.25 GPa, one can see that the superconducting transition temperature *T*_*c*_ is clearly increased from 3.8 K to 5.8 K with the pressure increased to about 1.74 ± 0.05 GPa, then *T*_*c*_ drops down slightly with further increase of pressure. We can see a sudden broadening of resistive transition at 2.25 ± 0.05 GPa, this may arise from the possibility that the pressure transmitting to different parts inside the sample is inhomogeneous at that pressure, or due to some other unknown reasons. Here the *T*_*c*_ value was determined at the temperature with 50% of the normal state resistivity *ρ*_*n*_. We should notice that, the *T*_*c*_ value determined by different methods may be slightly different. For example, the zero resistivity temperature *T*_*c*_*(zero)* may roughly correspond to the onset transition point in the magnetic susceptibility measurements. That is why the *T*_*c*_ value determined in present paper is slightly higher than that determined in ref. 23. The enhancement ratio of *T*_*c*_ versus pressure, i.e. *dT*_*c*_*/dp*, is about 1.11 K/GPa. This value is quite big and very surprise to us, since it is comparable to that in some unconventional superconductors^21^,^22^.

**Figure 2:**
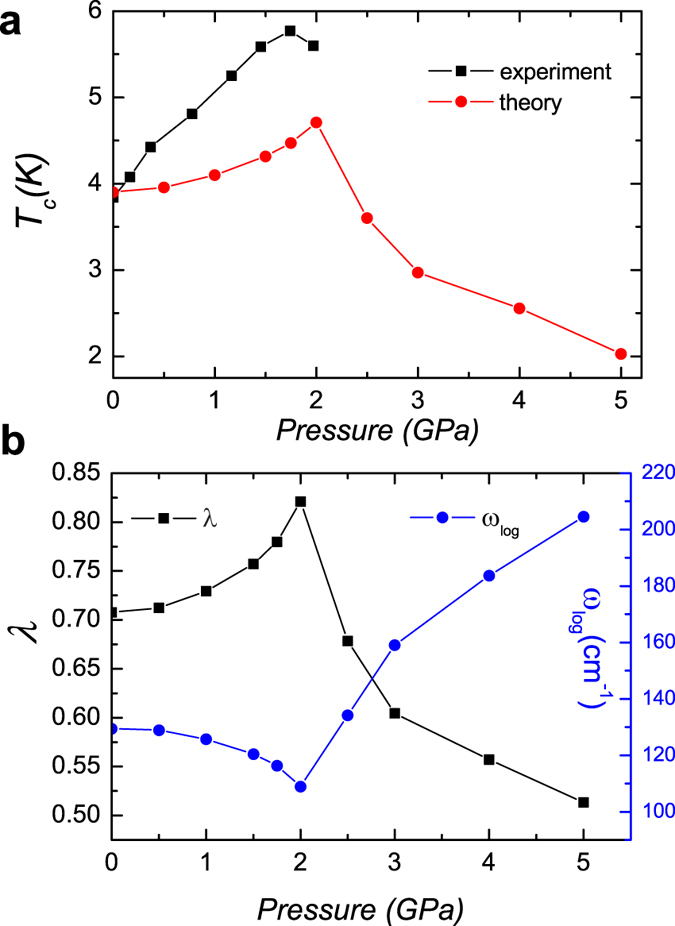
Pressure enhanced superconductivity and theoretical calculations. (**a**) Transition temperature *T*_*c*_ versus pressure for the LaRu_2_P_2_ single crystal obtained by the electrical resistivity measurement (black squares). Theoretical calculation results (red circles) are presented in the same graph for comparison, which is based on the McMillan’s theory for the case of strong electron-phonon coupling. (**b**) First principle calculation values of the maximum phonon frequency *ω*_log_ and the electron-phonon coupling constant *λ*_*e−ph*_ under different pressures. The calculation is based on the Density Functional Theory, using ultrasoft pseudopotentials, Generalized Gradient Approximation (PBE) functionals.

At a pressure higher than 1.74 ± 0.05 GPa, the *T*_*c*_ seems getting lower, as shown in Fig. 2a. The *T*_*c*_ at 2.25 ± 0.05 GPa is not presented in this graph since we cannot precisely determine the *T*_*c*_ at that pressure because of the broadening of resistive transition. Since there is only one confirmed point at 1.97 ± 0.05 GPa above the pressure with a maximum *T*_*c*_, we are not sure whether there is a dome like *T*_*c*_*-p* phase diagram. This behavior is not contradicted, but slightly different from that in the previously reported AC susceptibility measurements with hydrostatic pressures^23^, which reveals also an enhanced *T*_*c*_, but superconductivity suddenly disappears above 2.1 GPa. In the magnetic susceptibility measurements^23^, above about 2.1 GPa, the magnetic susceptibility vs. temperature curve becomes very smooth with a very strange background. Therefore it is very difficult to judge whether the superconductivity is really absent or just invisible due to the huge unknown background. In order to understand the superconductivity mechanism, we performed *ab-initio* calculations for the electron-phonon coupling in the frame work of density functional perturbation theory^24^, details of the calculations were provided in the Method section and Supplementary Information. Based on the McMillan theory for strong electron-phonon coupling, *T*_*c*_ can be estimated as:


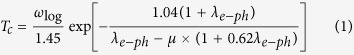


Here *ω*_log_ is the maximum phonon frequency and *λ*_*e−ph*_ is the electron-phonon coupling constant, *μ* is the Coulomb screening constant. Using the values of *ω*_log_ and *λ*_*e−ph*_ from the calculation for the pristine sample and taking *μ* = 0.12, we get a superconducting transition temperature *T*_*c*_ = 3.9 K. This is very close to our experimental value *T*_*c*_ = 3.8 K. This may indicate that the superconductivity in the sample at ambient pressure is induced by the electron-phonon coupling, which is consistent with the conclusion drawn previously^25^, which argues that the electron mass enhancement of LaRu_2_P_2_ mainly comes from electron-phonon coupling at ambient pressure. In order to understand the pressurized effect, we also did the calculations under pressures up to 5 GPa. With the calculated values of *ω*_log_ and *λ*_*e−ph*_ under different pressures, as shown in Fig. 2b, we do find a non-monotonic change of *ω*_log_ and *λ*_*e−ph*_ at a pressure of about 2 GPa. However, if we input all these calculated quantities into the McMillan’s formula (equation 1), we only get a slight enhancement of *T*_*c*_, but much weaker than that observed in the experiment. This suggests that the enhancement of superconducting transition temperature here cannot be interpreted as purely due to the conventional phonon mediated pairing.

**Figure 3:**
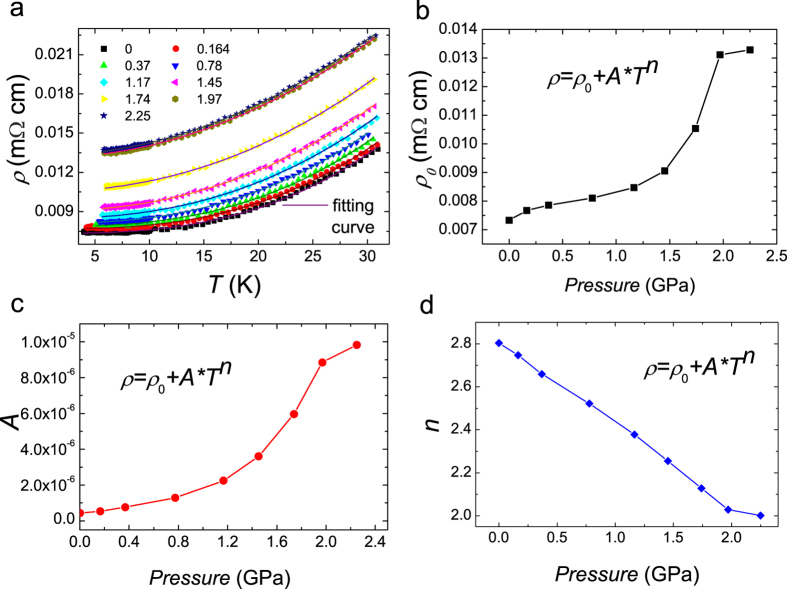
Fitting to the resistivity data under different pressures. (**a**) The electrical resistivity data and the fitting results under various pressures as shown by symbols and solid lines, respectively. The data of resistivity versus temperature is fitted in the temperature range of just above *T*_*c*_ to about 30 K with the general formula ρ = ρ_0_ + *AT*^*n*^, here *ρ*_*0*_ represents the residual resistivity due to the impurity scattering. The obtained coefficient *ρ*_*0*_, *A*, *n* under different pressures are presented in (**b**–**d**) respectively.

In order to get a deeper insight of the pressure induced enhancement of superconductivity, we take a look at the normal state resistivity under a pressure. The experimental data and the fitting results are presented in Fig. 3a as symbols and solid lines, respectively. We fit the data of *ρ* vs. *T* in the temperature range from just above *T*_*c*_ to about 30 K with the general formula (equation 2). Here *ρ*_*0*_ represents the residual resistivity due to the impurity scattering. According to the Matthiessens’s rule, the composed resistivity can be written as:


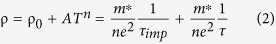


Here m^*^ is the effective mass when the quasiparticles are moving cross the lattice, *n* is the effective charge carrier density, *1/τ*_*imp*_ is the impurity scattering rate which is positively related to the impurity density *n*_*imp*_, *1/τ* is the scattering rate with the lattice. From the fitting results shown in Fig. 3b,c,d, one can see that, 
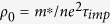
 increases with pressure and almost doubles at a pressure of 1.97 ± 0.05 GPa, then it turns to flatten off at higher pressures. The pre-factor *A* is quite complicated, it is related not only to the effective mass m^*^, but also to the electron-phonon coupling in a complex way. Therefore the increase of residual resistivity or *ρ*_*0*_ may be explained as the increase of m^*^, since m^*^ = *m*_0_(1 + *λ*_*e−ph*_ + *λ*_*e*−*boson*_) with *m*_*0*_ the bare mass of the electron, *λ*_*e*−*boson*_ is the extra electron-boson coupling strength in addition to the conventional electron-phonon coupling. In this simple argument, we can reasonably expect that 1 + *λ*_*e−ph*_ + *λ*_*e*−*boson*_ increases for about *two times* when the pressure is increased from zero to 1.74 ± 0.05 GPa. In Fig. 3d, we present the pressure dependence of the power exponent *n* which decreases from about 2.8 to 2.0 with the pressure enhanced from zero to 1.74 ± 0.05 GPa. This is consistent with the picture that extra electron-boson coupling sets in and induces the crossover from the conventional electron-phonon coupling to a moderate correlation effect. In the simple phonon scattering picture, a power law of 1/*τ* ∝ *T*^*n*^ (n = 3~5) was predicted in the low temperature region^26^. With the involvement of correlation effect, the power exponent *n* will be lowered down to 2, which is the exponent predicted in the Fermi liquid picture^27^. Therefore the evolution of *ρ*_*0*_, *A* and *n* with pressure can be self-consistently explained. This also explains why *T*_*c*_ is increased much faster than that predicted by the picture with simple phonon mediated pairing.

**Figure 4:**
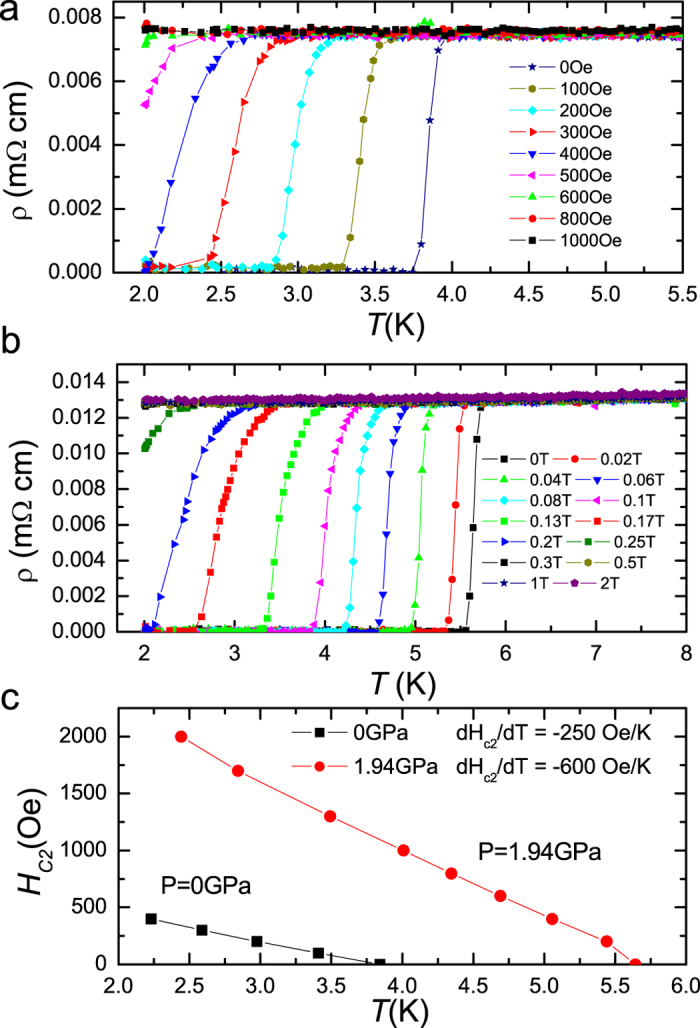
Resistive transitions and upper critical fields under ambient pressure and 1.94 GPa. (**a,b**) Temperature dependence of electrical resistivity for LaRu_2_P_2_ under various magnetic fields perpendicular to the basal plane at ambient pressure and 1.94 ± 0.05 GPa, respectively. (**c**) Temperature dependence of the upper critical field for the LaRu_2_P_2_ single crystal under ambient pressure and with a pressure of 1.94 ± 0.05 GPa, as shown by black squares and red circles, respectively. The transition temperature is obtained from Fig. 4a,b, using the 50%*ρ*_*n*_ criterion.

In order to give support to the picture mentioned above, we measured the temperature dependence of the upper critical field of the sample under ambient and a pressure of 1.94 ± 0.05 GPa. In Fig. 4a,b, we show the resistive transitions of the sample under these two states at different magnetic fields. We determined *T*_*c*_ at different magnetic fields using the 50%*ρ*_*n*_ criterion and present the data in Fig. 4c. To determine *H*_*c2*_*(T)*, one usually should not use the criterion of zero resistance since otherwise the vortex motion will be involved. It is clear that not only the *T*_*c*_ value is increased, the slope *−dH*_*c2*_*/dT* changes from about 250 Oe/K at ambient pressure to about 600 Oe/K at 1.94 ± 0.05 GPa. According to the Ginzburg-Landau theory, near *T*_*c*_ it was estimated that^28^ 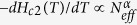 (*α* = 0.5~1) with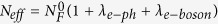, the effective density of states (DOS) with the total electron-boson coupling constant *λ*_*e*−*ph*_ + *λ*_*e−boson*_, where 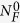 is the bare DOS at the Fermi energy. The increase of *−dH*_*c2*_*/dT* with pressure is very consistent with our previous conclusion that the enhancement of superconductivity is actually induced by the involvement of some extra electron-boson coupling which makes the system change from conventional electron-phonon dominated to moderate correlation governed Cooper pairing.

## Discussion

**Figure 5:**
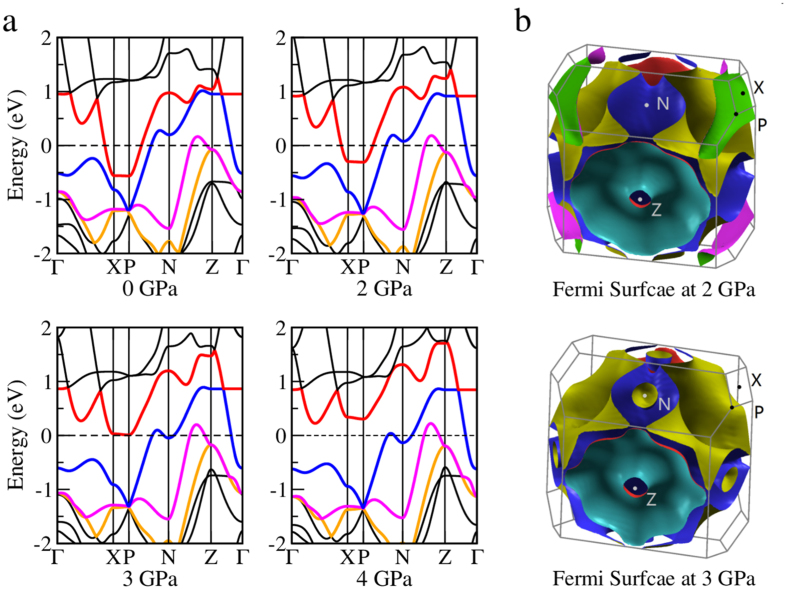
Band structures and Fermi surfaces under different pressures. (**a**) Change of the electronic band structure of LaRu_2_P_2_ (I4/mmm) with calculated lattice parameters from 0 GPa to 4 GPa; Four bands close to Fermi level are marked with different colors. The bands depicted with red and blue cross the Fermi level at about 3.0 GPa. (**b**) Change of the Fermi surface with calculated lattice parameters at 2.0 GPa and 3.0 GPa.

**Figure 6:**
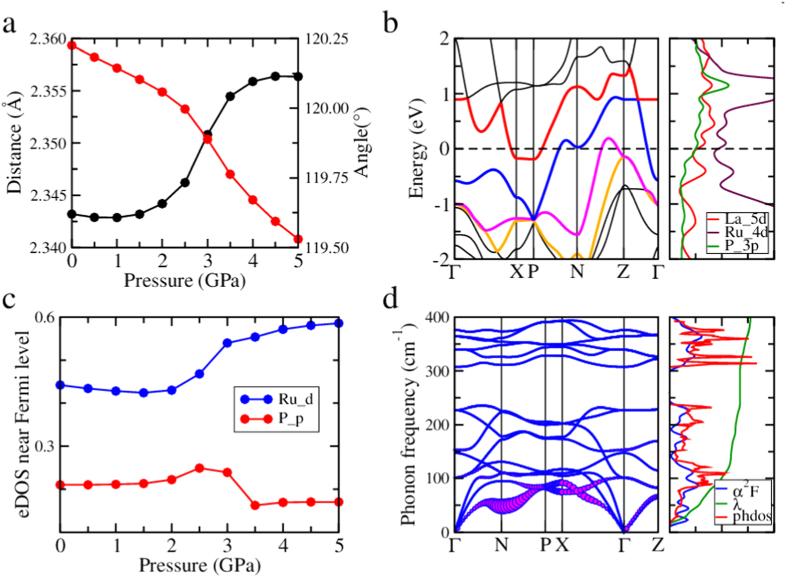
Theoretical calculations under different pressures. (**a**) Calculated values of Ru-P bond length (red line) and P-Ru-P angle (black line) in the RuP_4_ tetrahedron vs. pressure; An obvious enhancement of the Ru-P bond length (and reducing of the P-Ru-P angle) can be found at the pressure from 2 to 3.5 GPa. (**b**) The electronic band structure and DOS of LaRu_2_P_2_ (I4/mmm) with calculated lattice parameters at 2.5 GPa; The band lying on Fermi level between X and P (black) at 2.5 GPa are mainly contributed by P *3p(p*_*z*_) and Ru *4d(d*_*xz*_ + *d*_*yz*_) orbit. Panel on the right shows three partial DOS which make main contributions to the total DOS. (**c**) Calculated electronic DOS near Fermi level vs pressure, presents an increasing of Ru *4d* orbit and a dropping of P *3p* orbit, corresponding to (**a**,**d**) Calculated phonon dispersions at 2 GPa; the size of the bubble represents the electron-phonon interaction magnitude; phonon DOS and the integral value of electron-phonon coefficient *λ* are also shown on the right panel.

In the following we try to get some insights based on our *ab-initio* calculations. The band structures and the Fermi surfaces at ambient pressure are presented in Fig.5a and in the supplementary information, they are consistent with the previously published calculated and experimental results^25^,^29^. We relax the structure under different pressures and find a clear structural change upon pressure. As shown in Fig. 6a, the change of the Ru-P bond length within the Ru-P conducting layer exhibits an unusual step-wise feature at pressures of 2.0–3.5 GPa. This is accompanied by a slight closing of the P-Ru-P angle in the RuP_4_ tetrahedron and the slope for the change of the angle also varied a bit in the same pressure range. We can also find an obvious change on the electronic structures level. As we can see from Fig. 5a and Fig. 6b, four bands depicted with red, blue, pink and orange construct a complicated Fermi surface together. One band mostly contributed from Ru *4d(d*_*xz*_ + *d*_*yz*_) and P *3p*_*z*_ (red) moves upward with pressure and across the Fermi level at about 3.0 GPa, meanwhile, another band mainly consisting of Ru *4d(d*_*xz*_ + *d*_*yz*_) orbital (blue) moves downwards and the small wave-like feature near the N point also crosses the Fermi level at the same pressure range. This blue band opens a small tunnel in the Fermi surface after 3.0 GPa, as shown in Fig. 5b. *This movement slightly reduces the slope of the bands across the Fermi level, which may be very essential to enhance the effective electron mass and induce a moderate correlation effect.* From the electronic DOS in Fig. 6c, one can also see a clear variation from 2 to 3.5 GPa. The change of the Ru atom is mainly originated from its *d*_*xz*_ + *d*_*yz*_ orbitals while the change of the P atom is mostly coming from its *3p*_*z*_ orbital. Fig. 6d shows the phonon spectra, phonon linewidth, phonon density of states, Eliashberg function *α*^2^*F,* electron-phonon coupling (EPC) constant *λ* calculated for 2 GPa. It seems that a phonon mode near the N point have relatively large phonon linewidth and good contribution to the EP coupling. As shown in the Supplementary Information, this mode slightly goes soft with pressure and reach the lowest frequency at 2.5–3 GPa and goes harder afterwards. The EP coupling constant reaches a maximum value of 0.80 at about 2 GPa, which is somehow larger than that of the iron-arsenide system. As mentioned before, with all these refined structural parameters, the significant enhancement of *T*_*c*_ versus pressure in LaRu_2_P_2_ cannot be interpreted purely by the conventional electron-phonon coupling, extra electron-boson coupling may have been involved in the formation of superconducting pairing. In iron based superconductors, the antiferromagnetic spin fluctuations^30^,^31^ have been argued to be the dominant role in inducing the pairing. Therefore the pressure can induce a sensitive change of superconducting transition temperature as well as the normal state properties. In the 1111 family of iron pnictide high-temperature superconductors REFeAsO_1−x_F_x_, the hydrostatic pressure seems to show a similarity between the trend of critical temperature vs hydrostatic pressure for different rare earth elements (RE)^32^. The authors of that paper argue that this may indicate the role of pressure on the competing interactions in 1111 iron pnictides. As far as we know, no investigations on successful chemical doping have been reported in the system LaRu_2_P_2_. If the extra electron-boson coupling is important to enhance the superconductivity, it would be very interesting to carry out more works with chemical doping in the present system.

Finally, we need to emphasize that we still cannot completely confirm where does the extra electron-boson coupling come from through only the transport measurement. There is another possibility, we call it the electron-rattler interaction scenario, arguing that the “rattling phonon mode”, if exists in this 122 system, may be responsible for the enhanced electron-boson coupling strength. To be more explicit, if we view a pair of Ru-P structure inverted to each other as a cage with a La ion rattling in it, this rattling vibration may be influenced by the pressure and account for the enhanced *T*_*c*_ through the electron-rattler interaction, as what happens in the β-pyrochlore oxides AOs_2_O_6_ system^33^. This is also a very interesting possibility, and we think it is very meaningful to carry out relevant research in the future to have a deeper insight into this 122 system.

In conclusion, enhancement of superconducting transition temperature has been discovered in LaRu_2_P_2_ by pressure, which cannot be explained by the McMillan’s theory. A self-consistent analysis on the pressure induced evolution of normal state resistivity and upper critical field suggests that some extra correlation electron-boson interaction is induced by applying the pressure, which may be the cause of the pressure enhanced superconductivity. The present work strongly suggests that LaRu_2_P_2_ may have some commonalities as the iron based superconductors in which the spin fluctuations are supposed to play some roles in forming the Cooper pairs. This will stimulate future studies on the interplay of superconductivity and magnetism in the Ru-based systems.

## Methods

### Sample growth and measurement techniques

The single crystals LaRu_2_P_2_ were grown by flux method, using polycrystalline LaRu_2_P_2_ as precursor. The starting materials La metal scraps (99%), Ru powder (99.9%, Alfa Aesar), and phosphor powder (99.9%, Alfa Aesar) were weighed in stoichiometric ratio and mixed together, put into an alumina crucible. All these procedures were done in a glove box filled with Ar atmosphere. The crucible was sealed in an evacuated quartz ampule and kept at 1000 °C for 24 hours. The sintered LaRu_2_P_2_ powder was mixed with Sn flux in the molar ratio 1:40 and loaded into an alumina crucible, which was sealed in an evacuated quartz ampule and kept inside a PID controlled furnace box. It was raised to 1100 °C at a rate of 60 °C/h and maintained for 4 days, then the temperature was reduced down very slowly to 750 °C at a rate of 1.5 °C/h. The Sn flux was centrifuged out at 750 °C before cooling down to room temperature. Some flux sticking to the crystal surface was dissolved in an aqueous solution of hydrochloric acid.

X-ray diffraction (XRD) measurements were performed on a Bruker D8 Advanced diffractometer with the Cu-K_α_ radiation. DC magnetization measurements were carried out with a SQUID-VSM-7 T (Quantum Design).Measurements of resistivity under pressure were performed up to 2.3 GPa on a physical property measurement system (PPMS-16 T, Quantum Design) by using a HPC-33 Piston type pressure cell with the Quantum Design dc resistivity and ac transport options. For the resistive measurements, silver leads with a diameter of 50 μm were glued to the LaRu_2_P_2_ single crystal in a standard four-probe method by using silver epoxy, and the sample was immersed in the pressure transmitting medium (Daphne 7373) in a Teflon capsule with a diameter of 4 mm. Hydrostatic pressure was generated by a BeCu/NiCrAl clamped piston-cylinder cell. The pressure upon the sample was calibrated with the shift in *T*_*c*_ of a high purity Sn sample by measuring the temperature dependence of resistivity.

### *Ab-initio* calculation

First-principles calculations are performed using the Quantum-ESPRESSO code^34^, ultrasoft pseudopotentials with the Perdew-Burke-Ernzerhof (PBE)^35^ Generalized Gradient Approximation (GGA) density functionals are employed, phonon and electron-phonon coupling calculations are carried out within density functional perturbation theory (DFPT) framework^24^. The cutoffs are 80 Ry for the wave functions and 800 Ry for the charge density. The self-consistent calculations are performed over a 12 × 12 × 12 k-point grid. A denser 24 × 24 × 24 grid is used for evaluating an accurate EP interaction matrix. Dynamical matrices and the electron-phonon coupling were calculated on a 4 × 4 × 4 q-point mesh.

## Additional Information

**How to cite this article**: Li, B. *et al.* Pressure Induced Enhancement of Superconductivity in LaRu_2_P_2_. *Sci. Rep.* **6**, 24479; doi: 10.1038/srep24479 (2016).

## Supplementary Material

Supplementary Information
