# Supplementary material for: Pressure Induced Enhancement of Superconductivity in LaRu2P2
Source: Sci Rep. 2016 Apr 18;6:24479. doi: 10.1038/srep24479 (PMC4834477; doi:10.1038/srep24479)

Supplementary Information for

Pressure Induced Enhancement of Superconductivity in $LaRu_2P_2$

Baoxuan Li*, Pengchao Lu*, Jianzhong Liu, Jian Sun†, Sheng Li, Xiyu Zhu & Hai-Hu Wen†

I. Characterization of the $LaRu_2P_2$ crystal

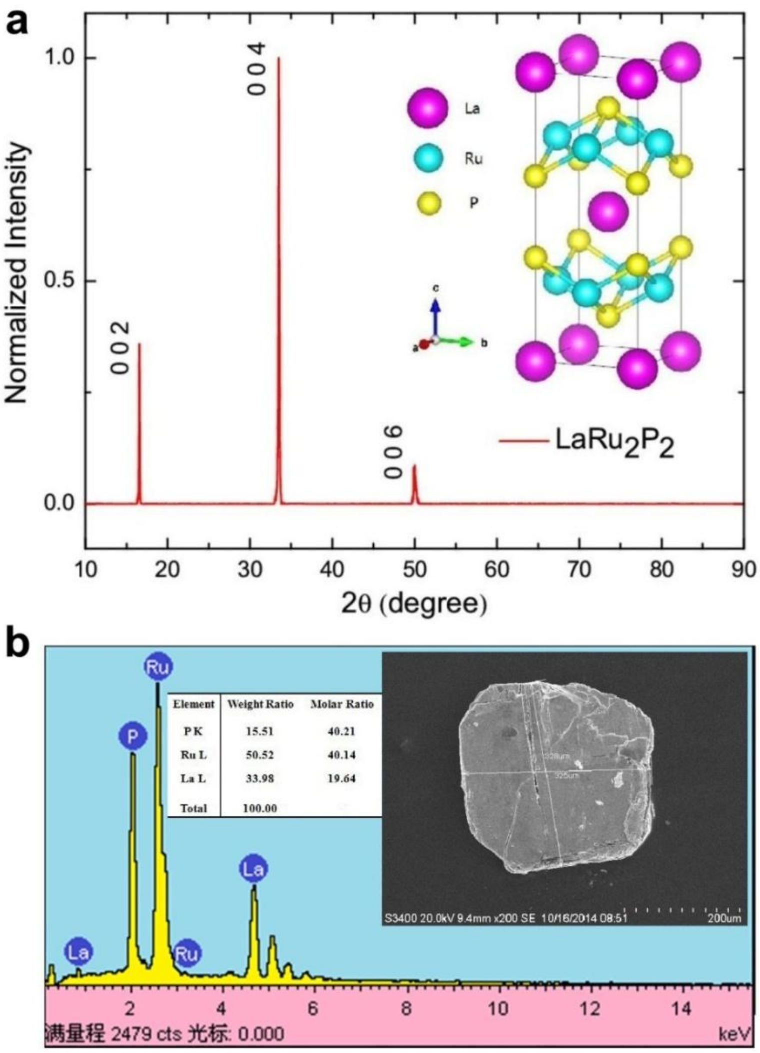

Figure S1 Characterization of the $LaRu_2P_2$ crystal. a, X-ray diffraction pattern for a $LaRu_2P_2$ single crystal. The inset gives the schematic show of the atomic structure of $LaRu_2P_2$, which adopts the tetragonal $ThCr_2Si_2$-type structure (I4/mmm space group). **b,** Energy Dispersive X-ray microanalysis pattern for $LaRu_2P_2$ single crystal. The inset shows the SEM photograph of the crystal with dimensions of about $325 \times 325 \times 40 \mu m^3$.

In Fig.S1a we present the X-ray diffraction pattern for the $LaRu_2P_2$ single crystal. It is clear that only the (0 0 l) reflections can be detected, and we can obtain the c-axis lattice constant c = 10.690Å from these peaks. Energy dispersive X-ray spectrum (EDS) measurements were performed at an accelerating voltage of 20kV and working distance of 10 millimeters by a scanning electron microscope (Hitachi Co.,Ltd.). Fig.S1b shows one set of the EDS result on a $LaRu_2P_2$ single crystal, the atomic ratio is very close to La:Ru:P = 1:2:2, probably with some La vacancies, as is shown in the table in Fig.S1b.

II. Ab-initio calculation on the pressure induced change of structural and band structures

1. Change of the lattice constants with pressure

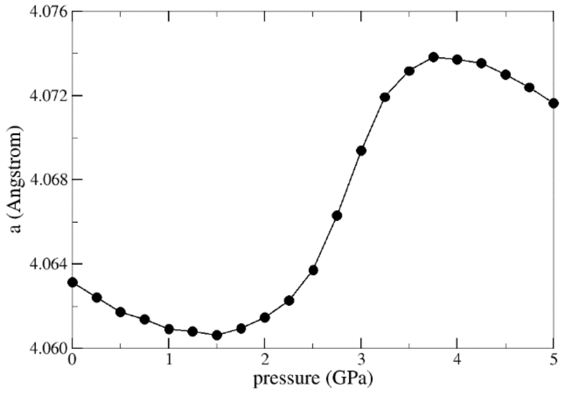

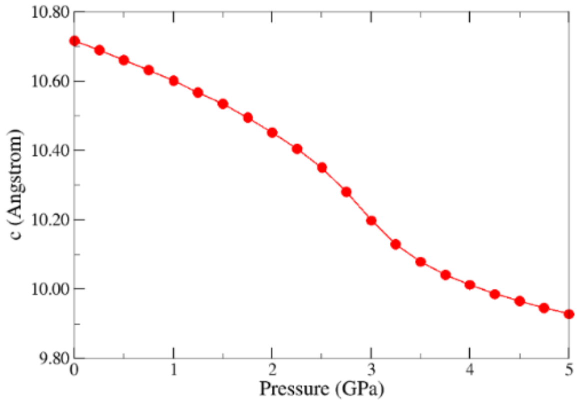

2. Change of the band structures with pressure

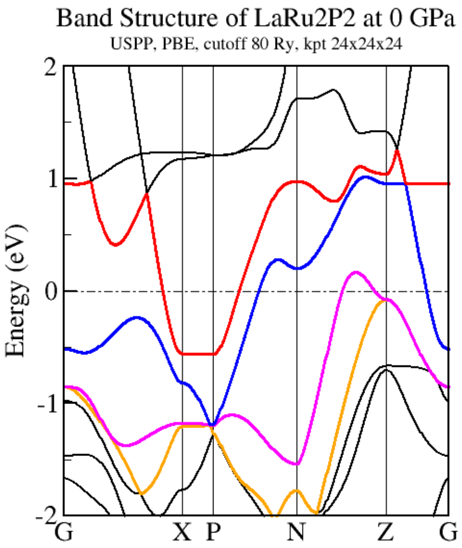

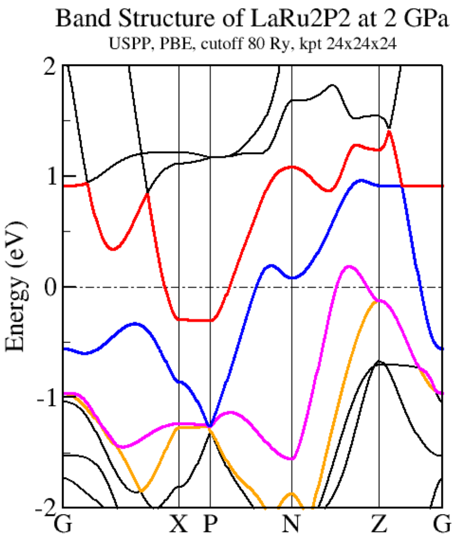

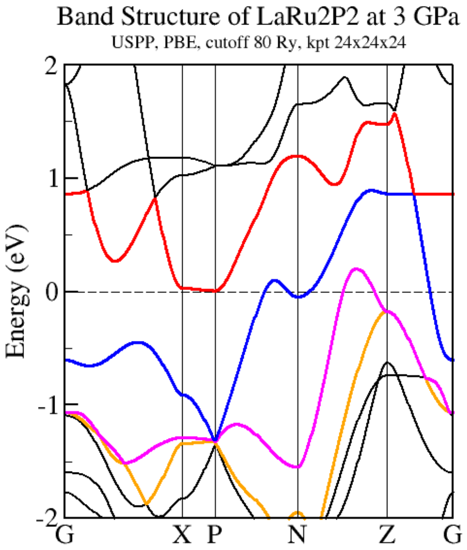

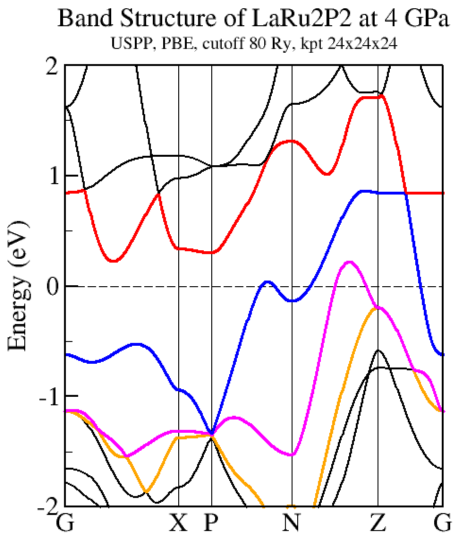

3. Change of the DOS with pressure

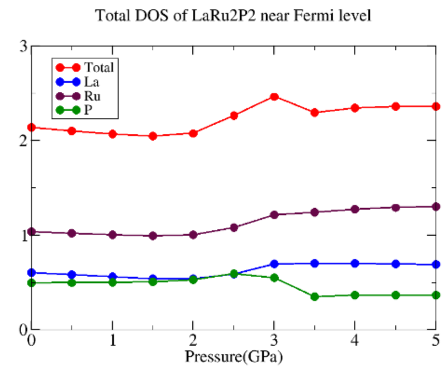

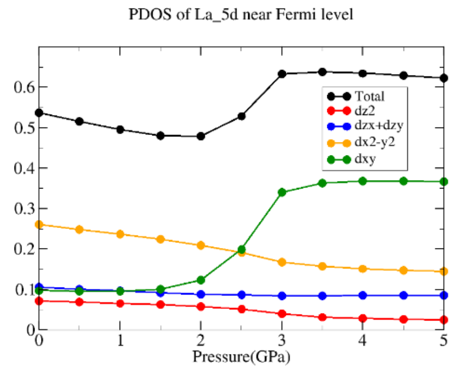

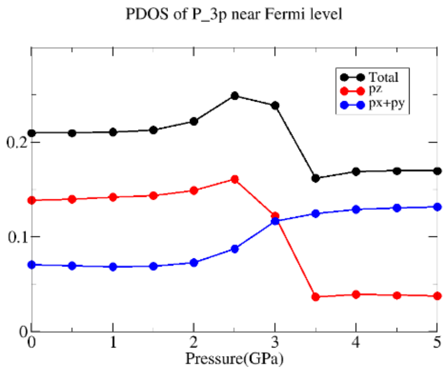

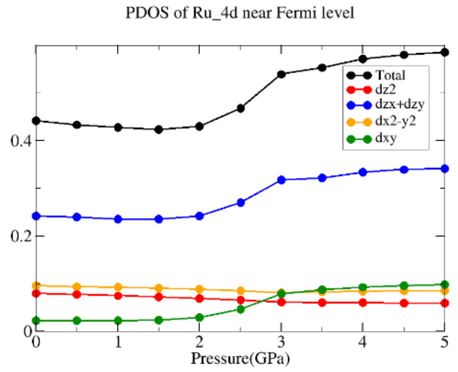

4. Change of the Fermi surface with pressure

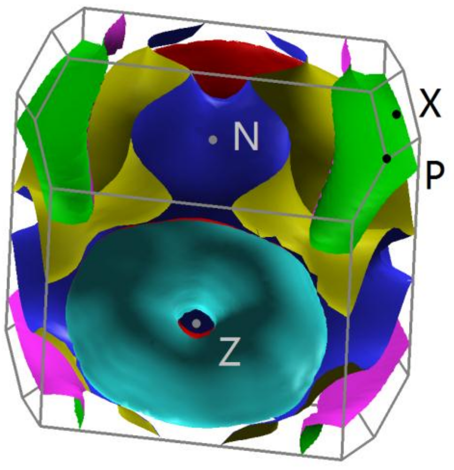

Fermi surface at 0GPa

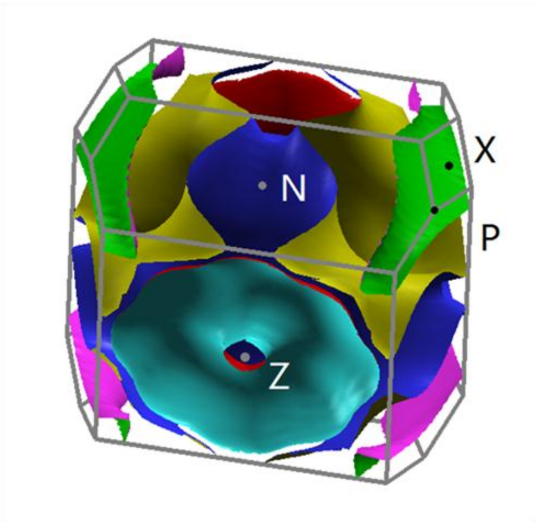

Fermi surface at 2GPa

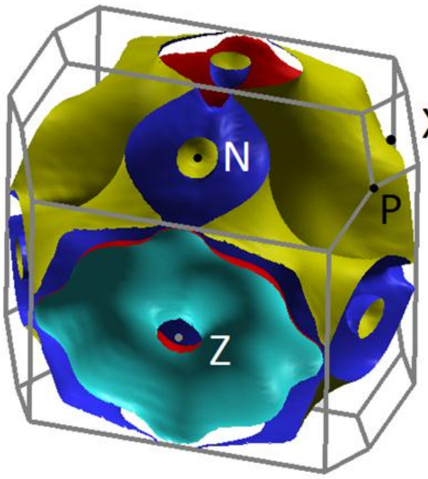

Fermi surface at 3 GPa

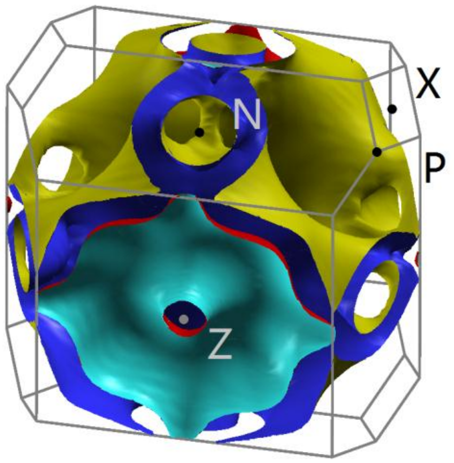

Fermi surface at 4GPa

5. Change of the bader charge with pressure

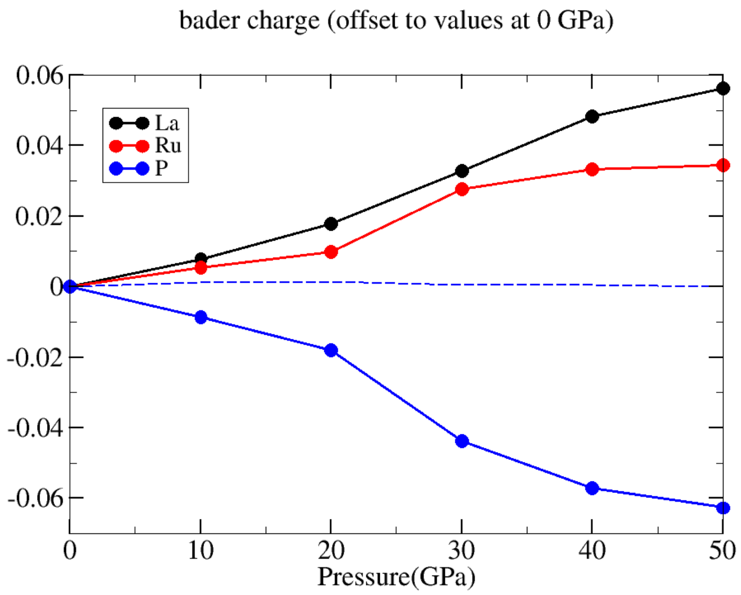

6. Change of the vibrational modes with pressure

Phononmodes at N point

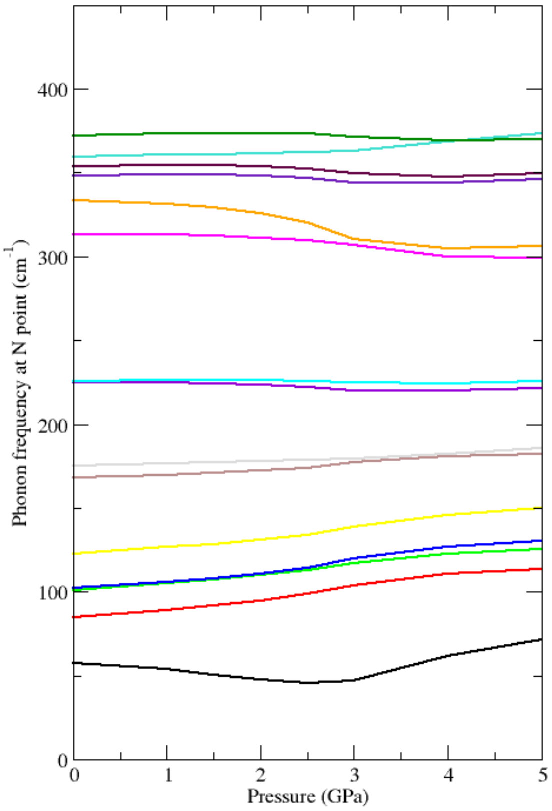

Supplement: Supplementary Information [file srep24479-s1.pdf]
